# Supplementary material for: Unveiling the Bioactive Potential of the Invasive Jellyfish Phyllorhiza punctata Through Integrative Transcriptomic and Proteomic Analyses
Source: Biomolecules. 2025 Aug 4;15(8):1121. doi: 10.3390/biom15081121 (PMC12383608; doi:10.3390/biom15081121)
Supplement: Supplementary file 1 [file biomolecules-15-01121-s001.zip › Suplementary Material/FileS3/PEPFOLD4/XP_065051124_10618/clusters.html]

 PEP-FOLD clusters


Download archive of all models  here   

|  |  |  |
| --- | --- | --- |
| # | file | sOPEP |
| # cluster 1 - Size: 17 |  |  |
|  | XP\_065051124\_10618-model1 | -13.774 |
|  | XP\_065051124\_10618-model1.1 | -13.774 |
|  | XP\_065051124\_10618-model1.2 | -13.774 |
|  | XP\_065051124\_10618-model1.3 | -13.774 |
|  | XP\_065051124\_10618-model1.4 | -13.774 |
|  | XP\_065051124\_10618-model1.5 | -13.774 |
|  | XP\_065051124\_10618-model1.6 | -13.6703 |
|  | XP\_065051124\_10618-model1.7 | -13.6703 |
|  | XP\_065051124\_10618-model1.8 | -13.6703 |
|  | XP\_065051124\_10618-model1.9 | -13.6703 |
|  | XP\_065051124\_10618-model1.10 | -13.6703 |
|  | XP\_065051124\_10618-model1.11 | -13.6703 |
|  | XP\_065051124\_10618-model1.12 | -13.6703 |
|  | XP\_065051124\_10618-model1.13 | -13.6703 |
|  | XP\_065051124\_10618-model1.14 | -13.6703 |
|  | XP\_065051124\_10618-model1.15 | -13.6703 |
|  | XP\_065051124\_10618-model1.16 | -13.6703 |
| # cluster 2 - Size: 5 |  |  |
|  | XP\_065051124\_10618-model2 | -13.7724 |
|  | XP\_065051124\_10618-model2.1 | -13.7724 |
|  | XP\_065051124\_10618-model2.2 | -13.6793 |
|  | XP\_065051124\_10618-model2.3 | -13.6793 |
|  | XP\_065051124\_10618-model2.4 | -13.6793 |
| # cluster 3 - Size: 39 |  |  |
|  | XP\_065051124\_10618-model3 | -13.6913 |
|  | XP\_065051124\_10618-model3.1 | -13.6913 |
|  | XP\_065051124\_10618-model3.2 | -13.6913 |
|  | XP\_065051124\_10618-model3.3 | -13.6913 |
|  | XP\_065051124\_10618-model3.4 | -13.6913 |
|  | XP\_065051124\_10618-model3.5 | -13.6913 |
|  | XP\_065051124\_10618-model3.6 | -13.6913 |
|  | XP\_065051124\_10618-model3.7 | -13.6913 |
|  | XP\_065051124\_10618-model3.8 | -13.6913 |
|  | XP\_065051124\_10618-model3.9 | -13.6913 |
|  | XP\_065051124\_10618-model3.10 | -13.6913 |
|  | XP\_065051124\_10618-model3.11 | -13.6913 |
|  | XP\_065051124\_10618-model3.12 | -13.6913 |
|  | XP\_065051124\_10618-model3.13 | -13.6913 |
|  | XP\_065051124\_10618-model3.14 | -13.6913 |
|  | XP\_065051124\_10618-model3.15 | -13.6913 |
|  | XP\_065051124\_10618-model3.16 | -13.6913 |
|  | XP\_065051124\_10618-model3.17 | -13.6913 |
|  | XP\_065051124\_10618-model3.18 | -13.6913 |
|  | XP\_065051124\_10618-model3.19 | -13.6913 |
|  | XP\_065051124\_10618-model3.20 | -13.6913 |
|  | XP\_065051124\_10618-model3.21 | -13.6913 |
|  | XP\_065051124\_10618-model3.22 | -13.6913 |
|  | XP\_065051124\_10618-model3.23 | -13.6751 |
|  | XP\_065051124\_10618-model3.24 | -13.6751 |
|  | XP\_065051124\_10618-model3.25 | -13.6751 |
|  | XP\_065051124\_10618-model3.26 | -13.6751 |
|  | XP\_065051124\_10618-model3.27 | -13.6751 |
|  | XP\_065051124\_10618-model3.28 | -13.6751 |
|  | XP\_065051124\_10618-model3.29 | -13.6751 |
|  | XP\_065051124\_10618-model3.30 | -13.6751 |
|  | XP\_065051124\_10618-model3.31 | -13.6751 |
|  | XP\_065051124\_10618-model3.32 | -13.6751 |
|  | XP\_065051124\_10618-model3.33 | -13.6751 |
|  | XP\_065051124\_10618-model3.34 | -13.6751 |
|  | XP\_065051124\_10618-model3.35 | -13.6751 |
|  | XP\_065051124\_10618-model3.36 | -13.6751 |
|  | XP\_065051124\_10618-model3.37 | -13.6751 |
|  | XP\_065051124\_10618-model3.38 | -13.6751 |
| # cluster 4 - Size: 7 |  |  |
|  | XP\_065051124\_10618-model4 | -13.6649 |
|  | XP\_065051124\_10618-model4.1 | -13.6649 |
|  | XP\_065051124\_10618-model4.2 | -13.6649 |
|  | XP\_065051124\_10618-model4.3 | -13.6649 |
|  | XP\_065051124\_10618-model4.4 | -13.6649 |
|  | XP\_065051124\_10618-model4.5 | -13.6649 |
|  | XP\_065051124\_10618-model4.6 | -13.6649 |
| # cluster 5 - Size: 17 |  |  |
|  | XP\_065051124\_10618-model5 | -13.6523 |
|  | XP\_065051124\_10618-model5.1 | -13.6523 |
|  | XP\_065051124\_10618-model5.2 | -13.6523 |
|  | XP\_065051124\_10618-model5.3 | -13.6523 |
|  | XP\_065051124\_10618-model5.4 | -13.6523 |
|  | XP\_065051124\_10618-model5.5 | -13.6523 |
|  | XP\_065051124\_10618-model5.6 | -13.6523 |
|  | XP\_065051124\_10618-model5.7 | -13.6523 |
|  | XP\_065051124\_10618-model5.8 | -13.6523 |
|  | XP\_065051124\_10618-model5.9 | -13.6523 |
|  | XP\_065051124\_10618-model5.10 | -13.6523 |
|  | XP\_065051124\_10618-model5.11 | -13.6523 |
|  | XP\_065051124\_10618-model5.12 | -13.6523 |
|  | XP\_065051124\_10618-model5.13 | -13.6523 |
|  | XP\_065051124\_10618-model5.14 | -13.6523 |
|  | XP\_065051124\_10618-model5.15 | -13.6523 |
|  | XP\_065051124\_10618-model5.16 | -13.6523 |
| # cluster 6 - Size: 4 |  |  |
|  | XP\_065051124\_10618-model6 | -13.6512 |
|  | XP\_065051124\_10618-model6.1 | -13.6512 |
|  | XP\_065051124\_10618-model6.2 | -13.5809 |
|  | XP\_065051124\_10618-model6.3 | -13.5809 |
| # cluster 7 - Size: 5 |  |  |
|  | XP\_065051124\_10618-model7 | -13.6413 |
|  | XP\_065051124\_10618-model7.1 | -13.6413 |
|  | XP\_065051124\_10618-model7.2 | -13.6413 |
|  | XP\_065051124\_10618-model7.3 | -13.6413 |
|  | XP\_065051124\_10618-model7.4 | -13.567 |
| # cluster 8 - Size: 2 |  |  |
|  | XP\_065051124\_10618-model8 | -13.6302 |
|  | XP\_065051124\_10618-model8.1 | -13.6302 |
| # cluster 9 - Size: 1 |  |  |
|  | XP\_065051124\_10618-model9 | -13.6274 |
| # cluster 10 - Size: 1 |  |  |
|  | XP\_065051124\_10618-model10 | -13.6274 |
| # cluster 11 - Size: 1 |  |  |
|  | XP\_065051124\_10618-model11 | -13.6169 |
| # cluster 12 - Size: 1 |  |  |
|  | XP\_065051124\_10618-model12 | -13.5573 |
|  |  |  |
